# Rare Variants in Autophagy and Non-Autophagy Genes in Late-Onset Pompe Disease: Suggestions of Their Disease-Modifying Role in Two Italian Families

**DOI:** 10.3390/ijms22073625

**Published:** 2021-03-31

**Authors:** Filomena Napolitano, Giorgia Bruno, Chiara Terracciano, Giuseppina Franzese, Nicole Piera Palomba, Federica Scotto di Carlo, Elisabetta Signoriello, Paolo De Blasiis, Stefano Navarro, Alessandro Gialluisi, Mariarosa Anna Beatrice Melone, Simone Sampaolo, Teresa Esposito

**Affiliations:** 1 Department of Advanced Medical and Surgical Sciences, 2nd Division of Neurology, Center for Rare Diseases and Inter University Center for Research in Neurosciences, University of Campania “Luigi Vanvitelli”, 80131 Naples, Italy; FILOMENA.NAPOLITANO@unicampania.it (F.N.); giorgiabruno990@gmail.com (G.B.); chiaraterracciano@hotmail.it (C.T.); giuseppina.franzese@unicampania.it (G.F.); elisabetta.signoriello@gmail.com (E.S.); paolodeblasiis@gmail.com (P.D.B.); marina.melone@unicampania.it (M.A.B.M.); Simone.SAMPAOLO@unicampania.it (S.S.); 2 Institute of Genetics and Biophysics “Adriano Buzzati-Traverso”, National Research Council, 80131 Naples, Italy; federica.scotto@igb.cnr.it (F.S.d.C.); Navarro_S@ukw.de (S.N.); 3 Neurology Unit, Guglielmo da Saliceto Hospital, 29121 Piacenza, Italy; 4 IRCCS INM Neuromed, Pozzilli, 86077 Isernia, Italy; nicole.palomba.89@gmail.com (N.P.P.); alessandro.gialluisi@gmail.com (A.G.); 5 Sbarro Institute for Cancer Research and Molecular Medicine, Center for Biotechnology, Temple University, Philadelphia, PA 19122, USA

**Keywords:** late-onset form of Pompe disease, skeletal muscle biopsies, modifiers factors, genetic modifiers, autophagy genes, whole exome sequencing

## Abstract

Pompe disease is an autosomal recessive disorder caused by a deficiency in the enzyme acid alpha-glucosidase. The late-onset form of Pompe disease (LOPD) is characterized by a slowly progressing proximal muscle weakness, often involving respiratory muscles. In LOPD, the levels of GAA enzyme activity and the severity of the clinical pictures may be highly variable among individuals, even in those who harbour the same combination of *GAA* mutations. The result is an unpredictable genotype–phenotype correlation. The purpose of this study was to identify the genetic factors responsible for the progression, severity and drug response in LOPD. We report here on a detailed clinical, morphological and genetic study, including a whole exome sequencing (WES) analysis of 11 adult LOPD siblings belonging to two Italian families carrying compound heterozygous *GAA* mutations. We disclosed a heterogeneous pattern of myopathic impairment, associated, among others, with cardiac defects, intracranial vessels abnormality, osteoporosis, vitamin D deficiency, obesity and adverse response to enzyme replacement therapy (ERT). We identified deleterious variants in the genes involved in autophagy, immunity and bone metabolism, which contributed to the severity of the clinical symptoms observed in the LOPD patients. This study emphasizes the multisystem nature of LOPD and highlights the polygenic nature of the complex phenotype disclosed in these patients.

## 1. Introduction

Glycogen storage disease type II (GSDII; Online Mendelian Inheritance in Man (OMIM) 232300; Pompe disease or acid maltase deficiency) is an autosomal recessive disorder caused by mutations in the acid alpha-glucosidase (*GAA*) gene encoding a lysosome enzyme that hydrolyses glycogen to glucose [1].

Mutations in *GAA* lead to a wide spectrum of clinical phenotypes, ranging from a severe infantile form, presenting with cardiomyopathy and muscular hypotonia, to a relatively mild, LOPD form characterized by a slowly progressing proximal muscle weakness, often involving respiratory muscles [1,2]. More recently, many additional symptoms associated with LOPD came to light: dysarthria and dysphagia, osteoporosis, scoliosis, sleep apnoea, small fibre neuropathy, hearing loss, impaired gastric function, urinary tract and anal sphincter involvement, pain and fatigue, as well as a risk of cardiac arrhythmia and cerebral and intracranial aneurysms [3]. These clinical findings emphasize the multisystem nature of LOPD.

In LOPD patients, the levels of GAA enzyme activity and the severity of clinical pictures may be highly variable among individuals, even in those who harbour the same combination of *GAA* mutations [4,5]. This indicates that there is no definite correlation between enzyme activity levels and severity of phenotype in LOPD patients [5,6,7,8,9,10,11,12,13,14,15]. This evidence leads to the hypothesis that the expression of *GAA* mutations could be modified by genetic background and non-genetic factors (life-style, nutrition and environment) [16,17]. To identify the modifier factors, which might affect the severity of symptoms of LOPD patients, studies on large informative families are advisable. Indeed, *GAA*-mutated family members have the closest possible genetic background and might better define the genotype–phenotype correlation, including a wide spectrum of clinical parameters.

In this manuscript, we report on a detailed clinical, morphological and genetic study of 11 adult LOPD siblings belonging to two large Italian families carrying compound heterozygous *GAA* mutations [18]. To better manage the wide spectrum of clinical and biochemical data, we calculated a severity index that was useful to simplify the genotype–phenotype relationships in both LOPD families.

## 2. Results

### 2.1. Clinical Evaluation of the LOPD Patients

**Figure 1:**
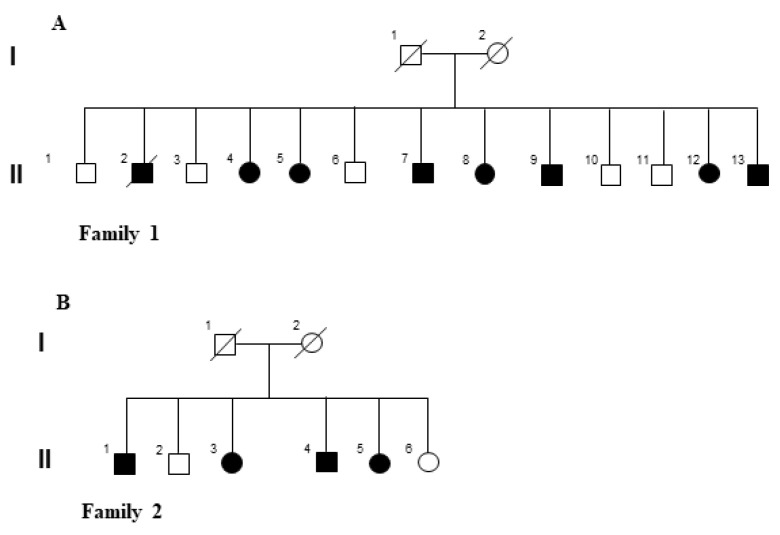
Pedigrees of the two Italian families. (**A**,**B**) Graphical representation of the two LOPD Italian families. The affected siblings are reported with filled symbols.

In this study, we focused our analysis on 11 LOPD siblings from two families (family 1: II-4, II-5, II-7, II-8, II-9, II-12, II-13; 4 females and 3 males; family 2: II-I, II-3, II-4, II-5; 2 females and 2 males) from Southern Italy (ages 50–67 years for family 1, and 41–51 years for family 2) (Figure 1A,B). Family 1 was already published [18]. To reduce the genetic heterogeneity, we included in this study only the patients of the second generation who carried the same combination of *GAA* mutations. Subjects II-1 and II-6 had a different *GAA* genetic background [18], a less severe phenotype than other siblings did, and they refused both ERT and WES analysis.

A complete description of all the clinical data collected in this study is reported in Table 1. All data were collected at baseline before ERT treatment. The immune response to ERT was assessed after the first two infusions and at three, six and twelve months of treatments, according to the ERT protocol, and the data shown in the Table 1 were those recorded at 12 months. 

Mean age at first symptoms was 41.7 years ± 6.7 standard deviation (SD) (range 29–53 years), with a higher average age at onset for females (43.5 ± 8.5 years) versus males (39.6 ± 3.2 years). The mean time lag between early symptoms and diagnosis was 12.5 ± 4.5 years. Analysis of motor function revealed mild reduced muscle strength in all patients, primarily affecting paraspinal and pelvic muscles, with manual muscle testing (MMT) mean score of 83.54% ± 8.61 and Gait, Stairs, Gower, Chair (GSCG) score of 11.09 ± 6.6. The 6-min walking test (6MWT) revealed a reduced physical functional capacity, achieving a distance of 241.66 ± 119 m for females and 279.2 ± 122.2 m for males.

The severity of muscle weakness varied between the patients regardless of gender and duration of the disease. A significant association between age at onset and muscle weakness was previously demonstrated in affected siblings of family 1 [18]. In accordance to this finding, looking at family 2 (Table 1), patients II-4 and II-5 showed worse motor capacity with earlier onset of symptoms (respectively at 38 and 29 years old) compared to other family members; in particular, subject II-5 was able to walk only with double support for a few meters (10 m at 6MWT). According to the Fatigue Severity Scale (FSS, Italian version), all our patients obtained a score higher than 5 (severe fatigue) [19]. The mean forced vital capacity (FVC) value in the upright position of all patients was 76.81% ± 21.76; the FVC was ≥80% of the predicted normal in 5 out of 7 siblings of family 1 (II-7, 8, 9, 12, 13) and only in II-1 of family 2 (Table 1). The other subjects analysed had reduced FVC upright values, which dropped in the supine position. However, patients II-4 and II-5 of family 2 (Table 1) were not able to perform spirometry in the supine position due the severe drop in respiratory performance, for which they needed non-invasive ventilation (NIV). These findings were suggestive of a restrictive respiratory pattern ranging from a mild to a moderately severe degree [20]. A meaningful correlation between respiratory impairment and age at onset was already reported in family 1 [18]; this is also true in family 2, in which the youngest members (II-4 and II-5) showed the most severe respiratory conditions compared to those of the other siblings (Table 1). Body mass index (BMI, kg/m^2^) was assessed since it represents a significant comorbidity in determining reduced motor capacity and worsening respiratory function. Although the patients with metabolic myopathies usually have normal or reduced BMI due to progressive muscle wasting, our patients showed an increased BMI mean value (27.74 ± 5.57) (Table 1). Furthermore, numerous studies demonstrated a high prevalence of osteoporosis in patients with LOPD [21]. To explore this aspect, we measured in all patients the circulating level of vitamin D and performed the analysis of bone mass density (BMD). The vitamin D levels were reduced in all subjects. In particular, patients II-12 and II-13 of family 1 and II-2 of family 2 (Table 1) showed a mean value of serum vitamin 25(OH)D of 19.25 ± 13.11, which denoted a status of insufficiency/deficiency. BMD was suggestive of osteopenia in most patients with reduced levels of vitamin D, whereas subjects F1-II-8 and F2-II-3 had normal T-score values despite vitamin D deficiency. In the patient II-4 of family 1, BMD indicated osteoporosis in accordance with her personal history of spontaneous vertebral collapse of two dorsal vertebrae. Furthermore, bone fractures occurred with low trauma and at a young age, with delayed healing, in patients F1-II-8 at 30 years old (proximal radius fracture) and in F2-II-5 at 42 years old (neck femur fracture). Additionally, LOPD is a predisposing condition to dilative arteriopathy and cerebral aneurysm formation [22], while structural cardiac alterations do not seem to be present when Pompe disease begins in adulthood [23]. We observed from brain magnetic resonance imaging (MRI) dilated intracranial internal carotid and basilar artery dolichoectasia in all siblings of family 1. Vascular anomalies in patient II-8, who carried a paramagnetic prosthesis, were confirmed through Angio-TC. Echocardiography revealed mild mitral prolapse in all subjects of family 1, except for II-13. Members of family 2 did not show any cerebral-vascular anomaly or cardiac valvular diseases. At the time of first evaluation of the patients, the GAA activity on Dried Blood Spot (DBS) was assessed, revealing a significantly reduced activity (mean values 0.56 ± 0.78 mol/h/L). Surprisingly, in subject II-4 of family 1, the dosage was in the normal range. A high number of lymphocytes containing Periodic Acid–Schiff (PAS)-positive granules were present on blood smears in the majority of the patients, with mean percentage values of 50.36% ± 21.36% (Table 1). Histological analysis of the muscle samplings detected a percentage of vacuolated fibres of 3.09% ± 5.02%, higher in the patients II-9 of family 1 (11.18%) and II-5 of family 2 (15.31%). Although the vacuolated fibres are representative of autophagy impairment in skeletal muscles, they do not seem to correlate with duration of the disease, age at biopsy nor with the residual GAA enzyme activity [18,24]. Finally, response to ERT and adverse reaction to therapy were considered. Specific serum IgG against rh-GAA ranged 850 to 6400 after one-year therapy. Patient II-5 of family 1 developed a severe adverse reaction after 18 months of ERT, disclosing diffuse cutaneous rash and shortness of breath during infusion. There were increased levels of specific serum IgG (51,000 mg/dL) and C3 and C4 fractions of the complement, while the C1 inhibitor was decreased (0.1 g/L) and the IgE antibodies against rh-GAA were absent. 

### 2.2. Generation of a Phenotype Severity Index (SI) for LOPD

To simplify the large amount of clinical results and define the relationship between clinical variability and genetic factors, we elaborated a phenotype SI. The minimum score for SI was 1, which indicates a diagnosis of LOPD without additional significant clinical signs, while the maximum SI score was 47. Results are reported in Table 2. The grade of severity for a single item was assigned: grade 0 indicated normal findings; age at onset (years old) was graded from 1 to 6 (grade 1: >50; grade 2: 49–40; grade 3: 39–30; grade 4: 29–15; grade 5: 14–10; grade 6: 9–1), while Manual Muscle Testing—Medical Research Council (MMT-MRC) (%) was graded from 0 to 4 (grade 0: 100%; grade 1: 99–90; grade 2: 89–80; grade 3: 79–70; grade 4: <69). 6MWT (m) severity score was different by gender (6MWT for females: grade 0: 480 ± 57; grade 1: 422–362; grade 2: 361–301; grade 3: 300–240; grade 4: 239–179; grade 5: 178–118; grade 6: <118; 6MWT for males: grade 0: 580 ± 44; grade 1: 535–476; grade 2: 475–395; grade 3: 394–315; grade 4: 314–234; grade 5: 233–150; grade 6: <150), whereas GSCG score was considered equally for both sex (grade 0: 4; grade 1: 5–8; grade 2: 9–15; grade 3: 16–21; grade 4: 22–27). We attributed a grade from 1 to 4 for FVC (%) (grade 0: > 80; grade 1: 79–70; grade 2: 69–60; grade 3: 59–50; grade 4: <50) as well as for Δ-FVC (%) (grade 0: <10; grade 1: 10–15; grade 2: 15–20; grade 3: 20–30; grade 4: >30). BMI (Kg/m^2^) score was determined according to the definition of a normal weight, overweight and obesity as previously described (grade 0: 19.5–24.9; grade 1: 25–29.9, grade 2: 30–35.9, grade 3: 36–40, grade 4: >40). Reduced vitamin D 25-OH serum levels (ng/mL) were classified in accordance to the common definitions of insufficiency and deficiency (grade 0: 30–100; grade 1: 20–30; grade 2: <20), and for the BMD were considered values of the T-score (g/cm^2^) of three different districts if suggestive of osteopenia (grade 1) or osteoporosis (grade 2). We assigned a grade of 1 to bone fractures (grade 0: no history of bone fractures; grade 1: presence of bone fractures), basilar artery dolichoectasia (grade 0: normal findings at brain MRI; grade 1: presence of BAD) and to mitral valve prolapse (grade 0: normal mitral valve; grade 1: presence of mitral valve prolapsed). Equally was attributed a score of 1 to the relief of serum IgG anti-rhGAA (grade 0: absence of IgG; grade 1: presence of IgG). 

For the FSS, all our patients obtained a score higher than 5 (severe fatigue) [19] and therefore the inclusion of this measure would not have changed the severity index calculation.

Moreover, since a clear relationship between disease severity and GAA activity in LOPD, as well as vacuoles in muscle fibres and in lymphocytes was not established yet [25,26], items related to autophagy impairment were not be considered in this index. Finally, the phenotype of the patients was divided into very mild (SI 1–10), mild (SI 11–20), moderate (SI 21–29), severe (SI 30–39), and very severe (SI ≥ 40).

As reported in Table 3, the clinical variables significantly associated with a worse severity score were global 6MWT (*p* = 0.03), FVC (*p* = 0.01), ΔFVC (*p* = 0.02), VDD (*p* = 0.01), BMD femoral neck (*p* = 0.03) and GSGC (*p* = 0.01). Considering the small sample size, 6MWT was analysed as global because the sex distribution was well distributed between the two subgroups. The association between 6MWT, GSGC, FVC, ΔFVC and the severity index score was also confirmed by Pearson correlation.

### 2.3. Comprehensive Genetic Analysis of the LOPD Patients

At the molecular level, the two families carried compound heterozygous mutations in *GAA*. Specifically, the 7 siblings of family 1 carried the two mutations c.118C > T (p.R40X) (reported as very severe in the Pompe Mutation Database) and c.2647-7G > A (p.N882fs) (potentially mild) [18], while the 4 siblings of family 2 carried the two mutations c.-32-13T > G (potentially mild) and c.1124G > T p.(Arg375Leu) (potentially less severe). Although the *GAA* c.2647-7G > A (p.N882fs) splicing variant of family 1 was associated to variable expression of the mutated allele [18], this mutation alone cannot explain the complex phenotype observed in these patients.

Considering that affected siblings belonging to the same family share the same *GAA* genomic region, we used the WES approach to search for other gene variants/mutations that might influence the severity of the LOPD phenotype. 

**Figure 2:**
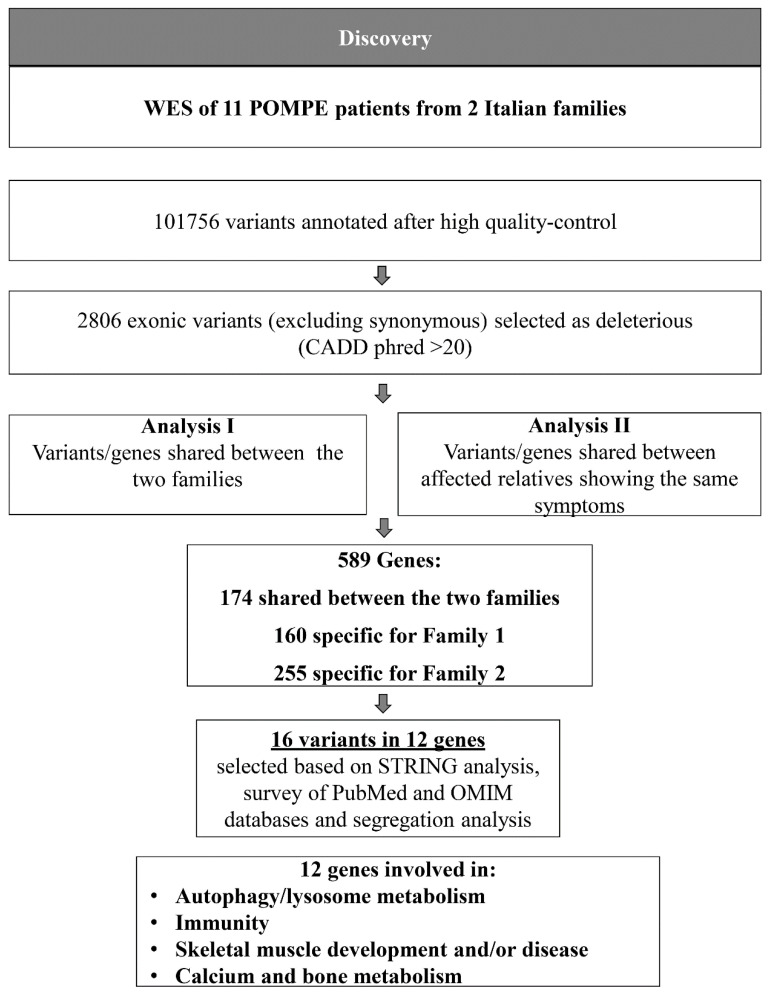
Pipeline of the analysis. The flowchart explains the analysis of WES data to identify the gene/variants shared between the two families (Analysis I) or between the affected relatives in each family (Analysis II). Functional annotation was performed with transcripts of the RefSeq and UCSC databases. MAF annotations were based on the 1000 Genomes project, Exome Variant Server, and the ExAC database. A CADD phred score > 20 was chosen to select deleterious variants.

We adopted a prioritization scheme to identify novel variants in modifier genes, similar to the approach taken in our recent studies (Figure 2) [27,28]. We annotated 101,756 high-quality variants from which we selected splicing variants (including splice acceptors and donor sites) and exonic variants, including non-synonymous variants (NSV), stop gain/loss and short coding insertions or deletions (indels; I) (16,230 variants); synonymous variants were excluded. Subsequently, we excluded the very common variants (minor allele frequency (MAF) ≥ 0.2) and selected the variants reported as highly deleterious (Combined Annotation Dependent Depletion (CADD) score ≥ 20) (2,806 variants), corresponding to those variants predicted to be amongst the 1% of the most deleterious variants in the genome. To dissect the different aspects of the disease manifestation, we performed dichotomization of the clinical signs as reported in the SI score (Table 2). Each patient was classified for each specific symptom as positive/negative or mild/severe. Subsequently, we performed two different analyses to identify the variants/genes shared between the two families (Analysis I, Figure 2) and the variants shared among relatives in each family (Analysis II, Figure 2). We selected 589 genes, including 174 shared between the two families, 160 specific to family 1 and 255 specific to family 2. To further reduce the number of variants, we performed a functional prioritization of the candidate genes using a STRING database analysis, as described in Material and Methods.

**Figure 3:**
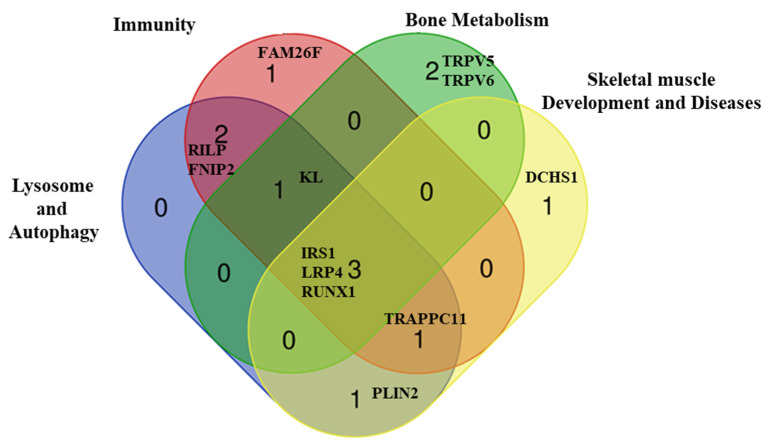
Venn diagram. Venn diagram showing the 12 modifiers genes involved across different interconnected pathways: lysosome–autophagy-related genes, genes involved in immunity, in bone metabolism and those related to the development of skeletal muscle and/or skeletal muscle diseases.

We identified 16 rare and deleterious variants (14 non-synonymous 1 non-frameshift insertion and 1 splicing) lying in 12 genes, involved across different interconnected pathways potentially deregulated in LOPD, including autophagy/lysosomal (*RILP*, *FNIP2*, *TRAPPC11*, *PLIN2*, *IRS1*, *KL*, *LRP4*, *RUNX1*), immunity (*RILP*, *FNIP2*, *TRAPPC11*, *IRS1*, *KL*, *LRP4*, *RUNX1*, *FAM26F*), bone metabolism (*TRPV5*, *TRPV6*, *IRS1*, *KL*, *LRP4*, *RUNX1*) and skeletal muscle development and/or disease (*TRAPPC11*, *PLIN2*, *IRS1*, *LRP4*, *RUNX1*, *DCHS1*) (Table 4 and Table 5 and Figure 3). Three out of the 12 genes, Rab Interacting Lysosomal Protein (*RILP*), Folliculin Interacting Protein 2 (*FNIP2*) and Perilipin 2 (*PLIN2*), were shared between the two families. *RILP* and *FNIP2* were associated to a SI score > 20 and *PLIN2* was associated with obesity in the two families. Polymorphic variants in the *DCHS1* gene, which causes dominant Mitral Valve Prolapse type 2 (MVP2) [26], were identified in all patients with MVP as single or compound heterozygous (patients II-5, II-7 and II-12), suggesting their role as MVP susceptibility factors. 

**Figure 4:**
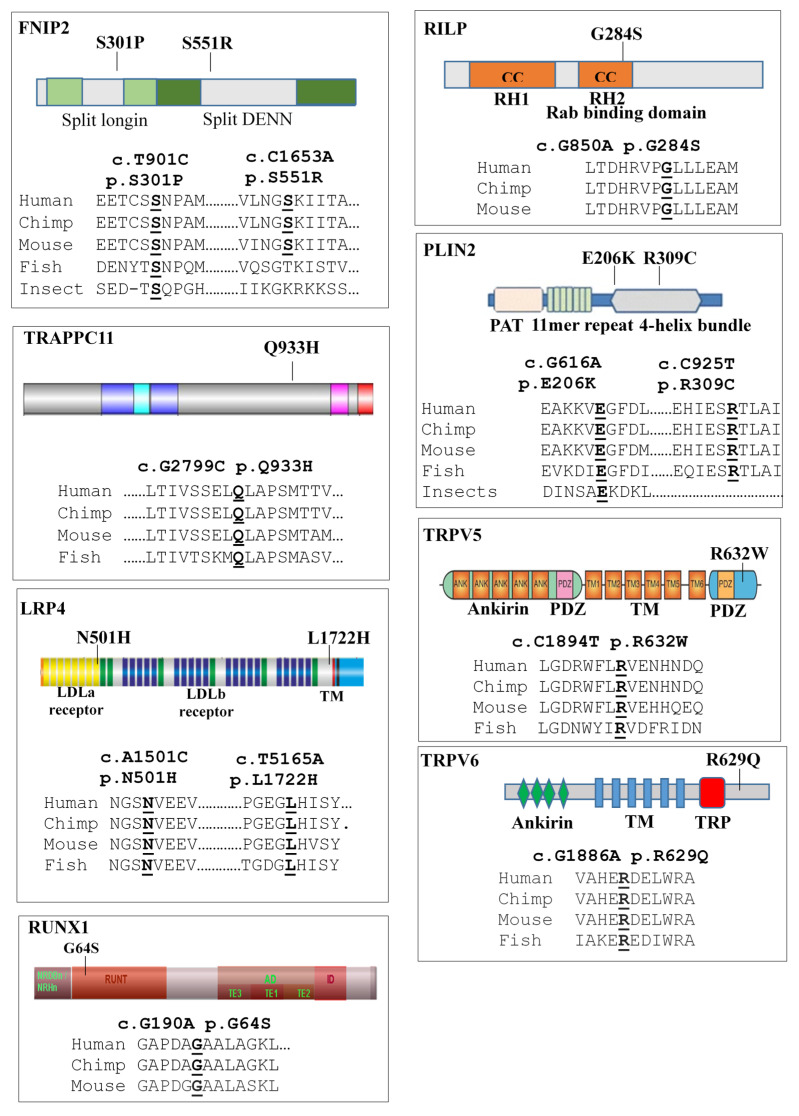
Evolutionary conservation study of the rare deleterious variants identified in the modifier genes. The affected residues are reported in bold underlined. For each gene, we reported the protein structure and the localization of the identified variant on protein domain.

Noteworthy, all the affected residues identified in the patients occupy functionally important amino acid positions, which are highly conserved amongst vertebrates (Figure 4), supporting the evidence in favour of their functional role. 

**Figure 5:**
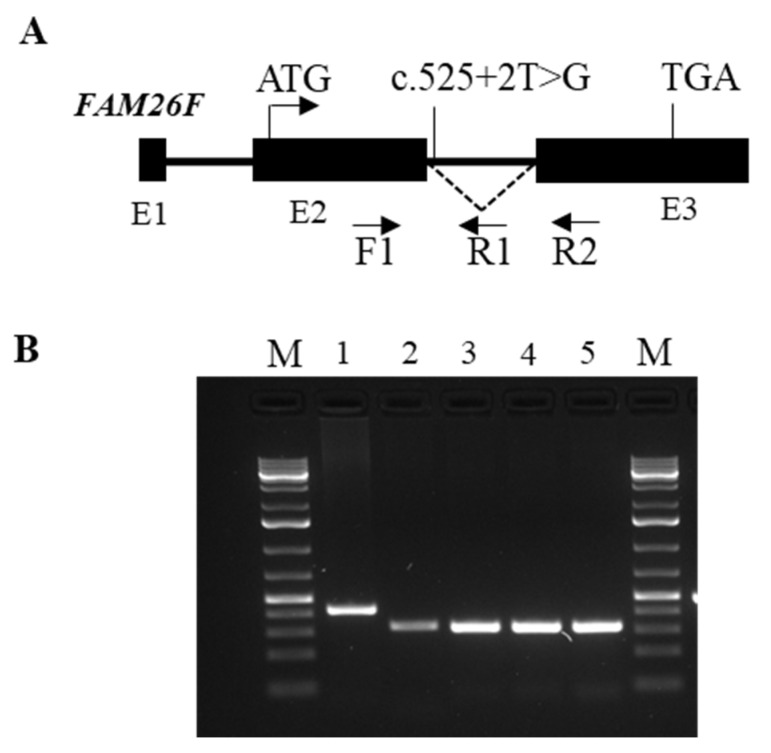
*FAM26F* gene structure and expression of the aberrant transcript. (**A**) Genomic structure of the human *FAM26F* gene. Exons are indicated by dark rectangles, introns with bold lines. The start site and stop codon are present in exons 2 and 3, respectively. The splicing mutation in intron 2 is indicated. Primers used in the expression analysis are indicated with arrows. (**B**) RT-PCR analysis. M: gene ruler 1 kb plus DNA ladder (Thermo Fisher Scientific, Waltham, MA, USA); lane 1: cDNA of patient II-5 amplified with primers F1-R1; lane 2: cDNA of patient II-5 amplified with primers F1-R2; lanes 3-5: cDNA of patients II-7, 9, 12 amplified with primers F1-R2. In patient II-5, the altered transcript carrying the unspliced intron 2 was amplified and a lower wild-type transcript was observed.

In the patient II-5 of family 1, who developed adverse response to ERT, we identified a splicing variant c.525 + 2T > G into the intron 2 of *FAM26F* gene, which codifies for a recently identified tetraspanin-like membrane glycoprotein that is involved in various immune modulating responses. A 100% probability was calculated by bioinformatics analysis that this variant completely disrupted the canonical donor splicing site into intron 2 of the *FAM26F* gene, suggesting that it was a deleterious change generating a frame shift (p.S174fs) and a predicted truncated protein of 224 amino acids. A reduced expression of the wild-type transcript was disclosed in patient II-5 combined with the presence of the aberrant spliced *FAM26F* transcript (Figure 5A,B).

Considering that the insufficiency/deficiency of vitamin D is the most important biochemical/clinical biomarker identified in our families, we investigated the allelic contribution in our patients of variants associated with low levels of vitamin D in genes involved in its metabolism. As reported in Table 6, we found in the two families an excess of CC alleles for the two variants rs2228570 (Vitamin D receptor (*VDR*) gene, c.T2C (p.M1T), also known as the FokI variant) and rs4588 (Vitamin D-binding protein (*GC/DBP*), c.C1307A (p.T436K)), which were reported as associated with low vitamin D levels in several populations [29].

### 2.4. Expression Studies

**Figure 6:**
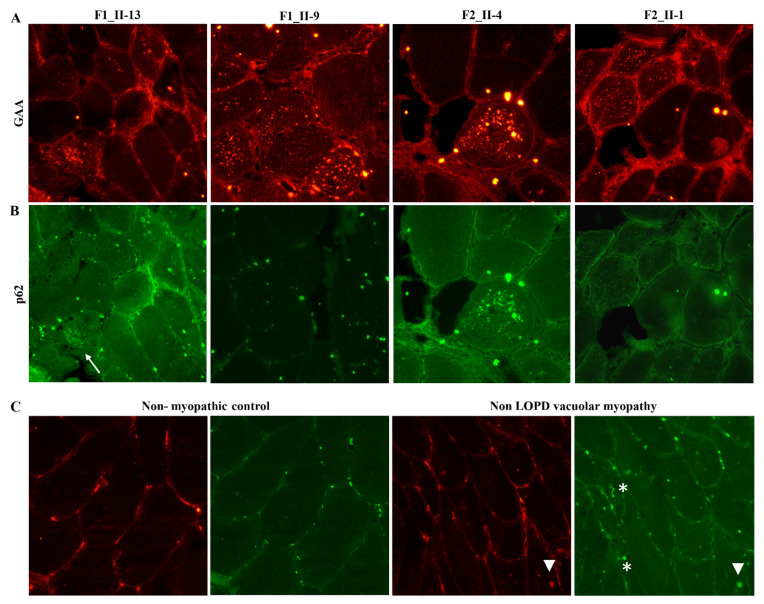
Skeletal muscle biopsies: immunofluorescence studies for GAA and p62 antibodies. (**A**) GAA/CY3 antibody marks the autophagic microvacuoles, which appear as red spots into the sarcoplasm of several fibres in all LOPD tissues. (**B**) Autophagic microvacuoles are intensely green when labelled by p62/FITC antibodies (impaired autophagy) or p62/FITC negative (active autophagy). (**C**) Non-myopathic skeletal tissue did not showed any GAA/p62-positive sarcoplasmic spots, while non-GAA vacuolar myopathy tissue presented with either GAA/p62-positive spots (arrowheads) or GAA-negative/p62 positive sarcoplasmic spots (white asterisks). Scale bar: 200 μm.

**Figure 7:**
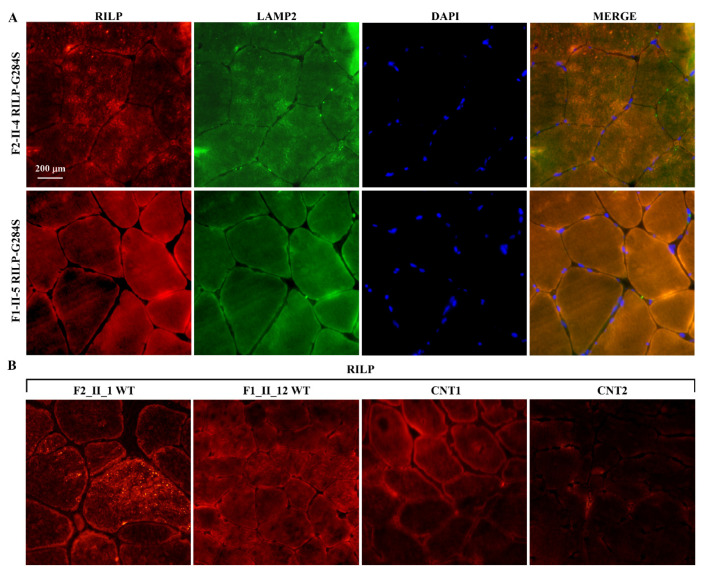
Skeletal muscle biopsies: immunofluorescence studies for RILP and LAMP2 antibodies. (**A**) In RILP mutated tissues the positive reaction for RILP/CY3 was visible both in the form of granular aggregates of variable extension and irregular profile located near the sarcolemma or scattered in the sarcoplasm (F2_II-4) and in the form of more or less extended subsarcolemmal linear reaction (F1_II-5). Immunofluorescence for LAMP2/FITC followed similar patterns of distribution but to a lesser extent and intensity. (**B**) The LOPD family members not mutated for RILP-positive RILP/CY3 granules appeared more intensely fluorescent than in mutated tissues with a similar subsarcolemmal and sarcoplasmic pattern of distribution. A non-LOPD vacuolar myopathy (CNT1) and a non-myopathic condition (CNT2) were used as external controls. In CNT1, a faint subsarcolemmal continuous signal was observed in the majority of fibres, while CNT2 showed almost no immunofluorescence. Nuclei were counterstained with DAPI (blue). Scale bar: 200 μm.

**Figure 8:**
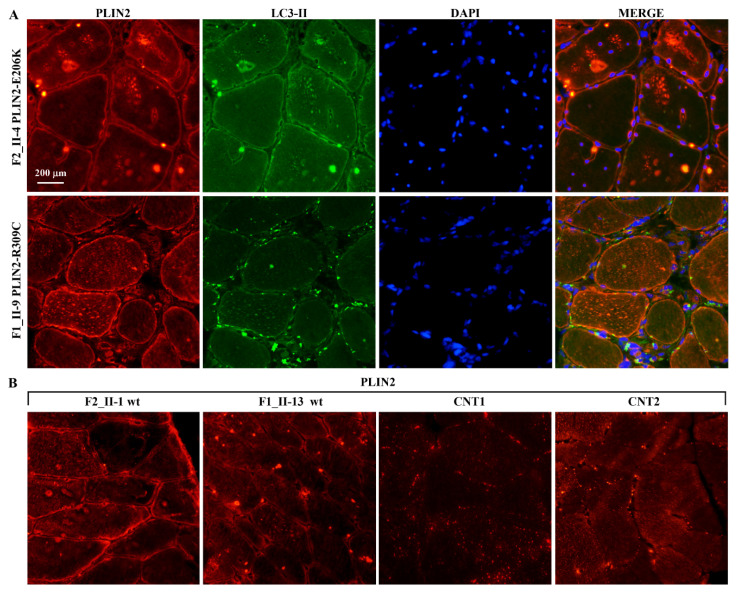
PLIN2 and LC3 immuno-localization on skeletal muscle tissues. (**A**,**B**) Continuous PLIN2/CY3 signal with variable intensity delineated the sarcolemma in mutated (**A**) and wild type (**B**) muscle fibres, while it appears faint and discontinuous in external controls (CNT1, CNT2). PLIN2/CY3 immunofluorescence marked numerous sarcoplasmic macro- and micro-vacuoles in the mutated muscles (**A**), which were less numerous in both wild type biopsies (**B**). External controls disclosed fine and scattered sarcoplasmic spots of PLIN2 positivity. Spots of LC3/FITC immunofluorescence were visible along the sarcolemma membranes and in overlap with only a few PLIN2-positive spots in mutated tissues (**A**). Nuclei were counterstained with DAPI (blue). Scale bar: 200 μm.

The immunofluorescence study on muscle biopsies showed that the GAA protein defined the profile of the muscle fibres and, in some of them, marked small sarcoplasmic spots, some of which were also p62 positive (Figure 6). p62, also known as Sequestosome 1 (*SQSTM1*), is involved in autophagy-dependent elimination of ubiquitinated proteins, and autophagy inhibition leads to the accumulation of p62-positive aggregates. RILP was expressed in the subsarcolemmal spots and at the level of the sarcoplasmic micro vacuoles in both RILP mutated and non-mutated biopsies of LOPD patients; however, the reaction appeared less intense in mutated tissues. In healthy subjects (CNT1), a faint subsarcolemmal continuous signal was observed in the majority of fibres, while CNT2 showed almost no fluorescent signal (Figure 7). The PLIN2 signal was intense and continuous at sarcolemma in mutated and wild type muscle fibres, while it appeared faint and discontinuous in external controls (CNT1, CNT2). PLIN2-positive sarcoplasmic macro and micro vacuoles were also evident in the mutated muscle, while in both the wild type and external control tissues smaller and scattered sarcoplasmic PLIN2-positive spots were seen (Figure 8).

**Figure 9:**
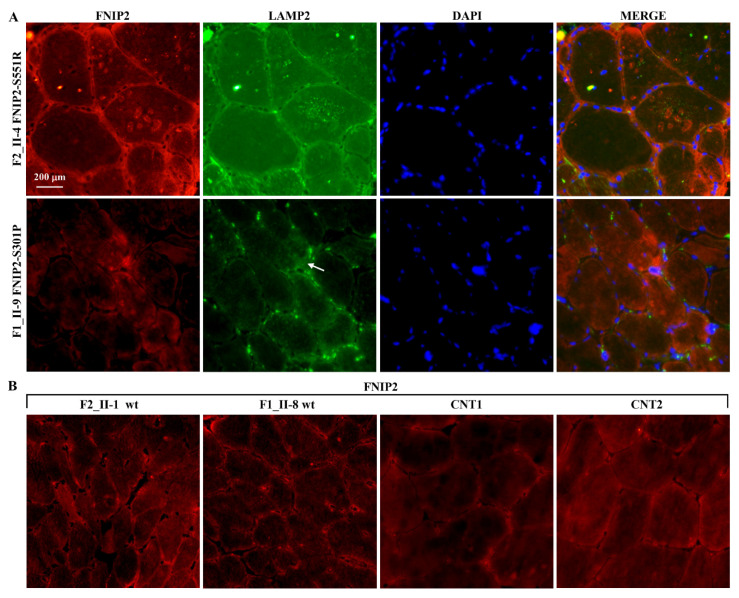
FNIP2 and LAMP2 immuno-localization on skeletal muscle biopsies. (**A**) FNIP2/CY3 expression was continuous and intense at the level of the sarcolemma membranes, and in the sarcoplasmic vacuoles and microvacuoles in FNIP2 [p.S551R] mutated biopsies, while more or less extended irregularly shaped spots of positive signals appeared at the edge in some fibres in (p.S301P) mutated biopsies. The LAMP2/FITC reaction was quite superimposable to that of FNIP2 in these biopsies. (**B**) A faint and diffuse FNIP2/CY3 positive reaction was observed at the sarcolemma and sarcoplasm of muscle fibres in both the wild type individuals and external controls (CNT1, CNT2). Nuclei were counterstained with DAPI (blue). Scale bar: 200 μm.

The FNIP2 reaction intensely labelled the vacuoles and micro vacuoles that also expressed the lysosome marker LAMP2 in the mutated biopsies. In the wild type as well as in external control tissues, the FNIP2 reaction marked weakly and diffusely the sarcolemma and the sarcoplasm of some fibres (Figure 9).

**Figure 10:**
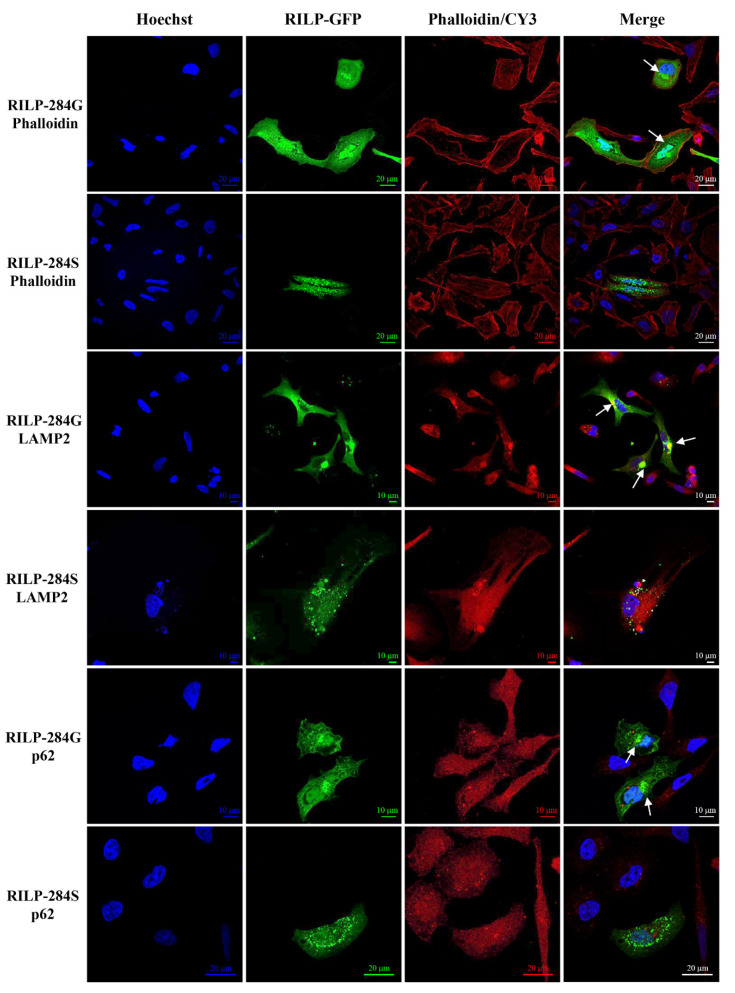
Immuno-localization of the RILP wt (RILP-284G-GFP) and mutant (RILP-284S-GFP) proteins in the HeLa cells wt and mutant. RILP proteins were fused to the green fluorescent protein (GFP), and LAMP2 and p62 proteins were stained using anti-LAMP2/CY3 and anti-p62/CY3. F-actin fibres were stained with rhodamin phalloidin and nuclei were stained with Hoechst. Slides were visualized with a Nikon Confocal Microscope A1R at 60× magnification.

The c.G850A (p.G284S) variant in *RILP* was associated with the most severe SI score in the two families. IF analysis in HeLa cells showed that the RILP284G protein accumulated near the nucleus (a strong signal intensity was observed) and co-localized with the LAMP2 antibody, suggesting lysosome and autophagosome localization. In contrast, a low signal intensity was observed for the RILP284S protein variant, associated with the SI score > 20, which was spread all over the cytoplasm and did not show a complete co-localization with LAMP2 and p62 antibodies (Figure 10). These data suggest that this variant might perturb the binding of the protein to lysosomes. 

The localization of the RILP wt is indicated by intense green spots localized near the nucleus. The LAMP2/CY3 reaction was quite superimposable to that of RILP. The RILP mutated protein is visualized as less intense green spots spread all over the cytoplasm. An incomplete co-localization with LAMP2 and p62 is highlighted. 

**Figure 11:**
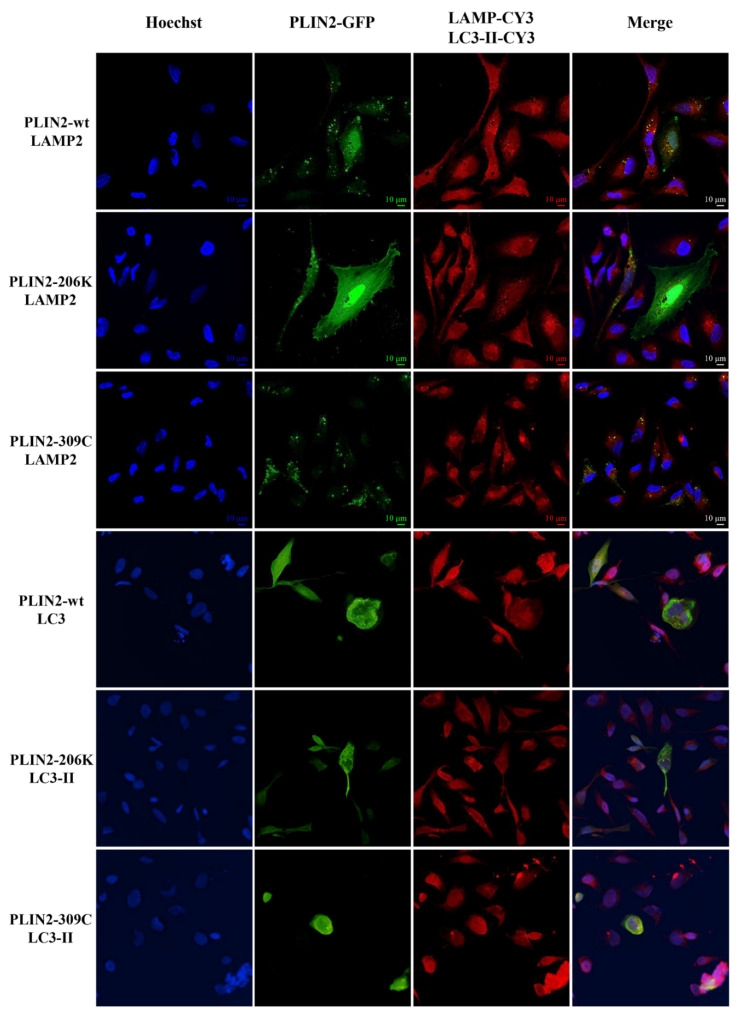
Immuno-localization of the PLIN2 wt (PLIN2-GFP) and mutant (PLIN2-206K-GFP) and (PLIN2-309C-GFP) proteins in the HeLa cells wt and mutant. PLIN2 proteins were fused to the green fluorescent protein (GFP), and LAMP2 and LC3 proteins were stained using anti-LAMP2/CY3 and anti-LC3/CY3. Nuclei were stained with Hoechst. Slides were visualized with a Nikon Confocal Microscope A1R at 60× magnification (LAMP2) and with Nikon Eclipse Ni-E 40x magnification (LC3-II). The localization of the PLIN2 wt and mutant proteins is indicated by green spots spread all over the cytoplasm. PLIN2 wt protein is localized in peripheral spots as well as weakly in the nucleus. The PLIN2-206K mutant protein is expressed intensely in the nucleus in several cells, while the PLIN2-309C mutant protein was observed only as peripheral spots.

In HeLa cells, the PLIN2 wt and mutated proteins (PLIN2206K and PLIN2309C) localized in the peripheral spots. The PLIN2 wt and PLIN2206K proteins also localized in the nucleus with different intensity (Figure 11).

In summary, we discovered a complex genetic background characterized by a set of rare variants in different genes worthy of future studies to confirm their role in the severity of the LOPD etiopathogenesis. These data highlights that the complexity of the LOPD phenotype is associated to a polygenic inheritance.

## 3. Discussion

Precision medicine is an emerging concept that stratifies the patients and their diseases by taking into account a wide array of individual data, including clinical and instrumental information, lifestyle, genetic background and individual drug responsiveness. 

In the present study, we described two LOPD families from South Italy manifesting with a complex spectrum of disease symptoms, determining their clinical heterogeneity also within the same family. 

The most frequent complaints were related to reduced muscle strength and fatigability, but some patients had to cope with respiratory insufficiency. These symptoms are usually the major contributors in developing severe complications during the disease course [30]. In this study, we generated a severity index score based on phenotypic manifestation of the disease and confirmed that impairment of motor capacity, assessed by GSCG scale as well as FVC and Δ-FVC values at baseline, were associated with a worse clinical outcome. Previous studies demonstrated that secondary factors might substantially influence the clinical course of the disease in LOPD patients, even if they carried the same *GAA* mutation [10]. In our cohort, alteration of bone metabolism, including vitamin D levels and BMD T-score of the femoral neck, showed a significant relationship with a higher value SI score. These results were supported by the evidence that low BMD is a frequent finding in patients with LOPD and may be causally related to decrease proximal muscle strength [21]. The essential role of vitamin D in muscle development and repair is well known, and the latest investigations demonstrated a genetic contribution of vitamin D to muscle functioning [31]. To our best knowledge, this is the first study demonstrating a significant association between low serum levels of Vitamin D and worse phenotype of LOPD patients. 

We performed WES analysis of the affected siblings and identified 16 deleterious variants in 12 genes *RILP*, *FNIP2*, *TRAPPC11*, *PLIN2*, *IRS1*, *KL*, *LRP4*, *RUNX1*, *FAM26F*, *TRPV5*, *TRPV6*, and *DCHS1*. These genes are involved in interconnected pathways, including autophagy/lysosome, immunity, bone metabolism and skeletal muscle disease-related genes, suggesting that rare deleterious variants in these genes or more generally alterations of these pathways might act to worsen the LOPD phenotype.

Autophagy is a dynamic cellular process associated with the pathogenesis of a wide range of human disorders, including LOPD [32,33]. This implies that the deleterious variants in autophagy-related genes may affect disease onset and progression [33]. Recently, it has been suggested that any mutation affecting the autophagosome–lysosome machinery reduces GAA activity as a consequence of the down regulation of GAA synthesis and maturation [34,35]. This implies that the concurrence in each patient of variants in two or more genes acting in various steps of the autophagy–lysosomal pathway might explain differences in the clinical expressivity of the *GAA* mutations. Remarkably, in this study, we identified five LOPD patients (F1-II4, F1-II5, F1-II9, F2-II4 and F2-II5) showing the highest severity index score, deleterious variants affecting important domains of RILP and FNIP2 proteins involved in lysosome cellular positioning and autophagy [36].

Rab interacting lysosomal protein (RILP) is a 45 kDa lysosomal protein that acts as a downstream effector of Rab7 in phago-lysosome formation and in the transport of the endocytic cargo to the lysosome [37]. Active Rab7 on the phagosomal membrane recruits RILP, which in turn promotes a dynein–dynactin association with the phagosomes, mediating their retrograde moving towards the microtubule-organizing centre (MTOC), where late endosomes and/or lysosomes were clustered [37,38]. Remarkably, the c.G850A (p.G284S) variant, identified in both families investigated in this study, lays into the Rab binding domain and alters a highly conserved residue of the protein, suggesting that this change might disturb the formation of the RILP–Rab complex. These bioinformatics predictions were supported by a different pattern of expression and localization observed in skeletal muscle of the patients carrying the mutated allele and in HeLa cells. The RILP-284S protein was not efficiently recruited at autophagosome–lysosome and remains spread all over the cytoplasm, suggesting a perturbation of the autophagic flux. 

Moreover, during nutrient insufficiency, the FLCN–FNIP complex modulates the formation of a FLCN–RILP–Rab complex for promoting the clustering of lysosomes around the nucleus [36].

Folliculin interacting proteins 2 (FNIP2) plays a crucial role in the regulation of the autophagic flux by modulating the formation of the FLCN–GABARAP complex [39]. Moreover, FLCN and FNIP proteins each contain both a longin and a differentially expressed in normal versus neoplastic cells (DENN) domain [40], which are implicated in the regulation of small GTPases and membrane trafficking. In fact, the FLCN–FNIP complex regulates both the Rag and Rab GTPase families [41,42], which in turn modulate the key mTORC1 signalling pathway and lysosomal distribution, respectively, in a manner dependent on amino acid availability [36]. Noteworthy, the two rare and deleterious variants identified in the two LOPD families alter the longin and DENN domains of the protein, suggesting that they might disturb the formation of this complex. 

Taken together these evidences lead to hypothesize that alteration of the genes involved in lysosome positioning and autophagy might influence lysosome metabolism, worsening the effect of the *GAA* mutations in the two investigated families.

Separate consideration needs to be given to patient II-5 of family 2, who manifested the most severe phenotype observed in our cohort of patients, characterized by severe muscle and pulmonary impairments (6MWT = 10, GSCG = 26, FVC = 36% and vacuolated muscle fibres = 15%). Remarkably, this patient inherited, together with the two mutations in *GAA* gene and the two rare and deleterious variants in the *RILP* and *FNIP2* genes, two compound heterozygous variants c.A1501C (p.N501H) and c.T5165A (p.L1722H), in the low-density lipoprotein receptor-related protein-4 (*LRP4*) gene. LPR4 is a major mediator of postsynaptic stability at the neuromuscular junction, a region critical for control of muscle contraction [43,44]. Recessive mutations in this gene are linked to congenital myasthenic syndrome (CMS17), a severe neuromuscular disorder associated to respiratory failure [43]. Respiratory related symptoms (dyspnoea on effort, reduced physical capacity and recurrent infections) and respiratory failure are often evident in the later stages of LOPD [45]. The two rare and deleterious *LRP4* variants identified only in this patient alter the highly conserved residues laying in important domains of the protein (Figure 4). In this context, we hypothesize that these variants, although reported as likely benign in the Global Variome shared LOVD database (https://databases.lovd.nl/shared/genes (accessed on 5 March 2021)), might have worsened the entire clinical picture of this patient. Moreover, she also shares with her brother II-4 a rare deleterious variant in *TRAPPC11*, whose mutations cause limb girdle muscular dystrophy 2S (LGMD2S), congenital muscular dystrophy (CMD) and dystroglycanopathy. Interestingly, this gene has a role upstream of autophagosome formation, by recruiting the ATG2B-WIPI4/WDR45 complex to pre-autophagosomal membranes, suggesting that the *TRAPPC11* variants might also perturb autophagy flux [46,47]. 

Two rare and deleterious variants disturbing the lipid-binding domain of the *PLIN2* gene segregated with obesity in the two families. *PLIN2* encodes the most abundant lipid droplet (LD)-associated protein in non-adipose tissue, and its expression correlates with intracellular lipid accumulation [48,49]. Recent studies reported that mutations in *PLIN2* affect lipolysis and are associated with reduced plasma triglyceride levels in humans [48], as well as with reduced insulin secretion and increased insulin sensitivity in obese Italian subjects [50]. Moreover, lipids in LDs were reportedly required for the induction of autophagy in yeast and served as the substrate of autophagosomes [51]. This aspect leads to the hypothesis of a link between obesity and autophagy impairment in LOPD patients. Here, we have demonstrated that RILP, FNIP2 and PLIN2 proteins localized at the sarcolemmal or sarcoplasmic level, with the same localization of markers of the autophagic-lysosomal system (LC3, LAMP2). Differences in the pattern of distribution in mutated versus wild type biopsies and even more of these versus external controls were evident. In particular, the different protein distribution between LOPD patients and the external control affected by vacuolar myopathy not linked to *GAA* mutations is very interesting. 

Recent studies reported that bone involvement is a common finding in Pompe disease, particularly in wheel-chair users, primarily characterized by low BMD and scoliosis. LOPD patients have a high risk of fragility fractures, chronic immobilization and muscle weakness and BMD reduction, particularly at the femoral neck, seems to be related to lower mechanical loading resulting from structural and functional impairment of the primary muscles involved in walking, such as hip flexors and knee extensors muscles [21,52]. Vitamin D plays a key role in the bone–muscle crosstalk [53]. In this context, its effects consist mainly of the maintenance of intracellular Ca2 + homeostasis that is of crucial importance for muscle contraction and bone metabolism. In the present work, deleterious variants in *IRS1*, *KL*, *RUNX1*, *TRPV5* and TRPV6 genes, involved in bone and mineral metabolism, were identified in family 1 [54,55,56,57,58,59,60,61,62,63,64,65,66].

In this context, we hypothesize that the load of polygenic rare and deleterious variants in genes closely related to the Ca2+ level and bone metabolism together with a perturbed autophagy, which plays an important role in bone remodelling, might explain the different spectrum of bone compromising in the family members.

Since 2006, ERT has been available for Pompe disease. ERT improved survival and motor function, normalized left ventricular cardiac hypertrophy and stabilized respiratory capacity [67]. However, such a treatment has brought to light the role of the immune response in abrogating the efficacy of the rhGAA treatment. Several patients are also cross-reactive immunological material negative (CRIM-) and develop high titre immune responses to ERT with rhGAA [68]. Thus, optimization of treatment for such patients includes development and utilization of strategies to prevent or eliminate immune responses, including modulating the immune system (prophylactic and therapeutic immune tolerance induction regimens) and engineering the enzyme to be less immunogenic and more effective.

In this study, we identified a deleterious variant in the *FAM26F* gene in the patient II-5 who manifested immuno-intolerance to rhGAA. In silico analysis showed that the splicing variant c.525 + 2T > G disrupted the donor splicing site of intron 2, producing a predicted truncated protein of 224 amino acids lacking the fourth transmembrane domain and of the cytosolic C-terminal tail of the protein [69]. The family with sequence similarity 26, member F (FAM26F), is a recently identified tetraspanin-like membrane glycoprotein involved in various immune modulating responses, including homophilic interactions and potential synapses between several immune cells, including CD4+, CD8+, NK, dendritic cells and macrophages. Several studies have demonstrated that FAM26F is highly crucial in determining/dictating the immune response of an individual, also in diseased conditions [70]. The ImmuNet bioinformatics tool predicts a high relationship confidence with the C1S gene that codifies a major constituent of the human complement subcomponent C1. 

We hypothesize that this variant might affect the expression of the FAM26F protein, thus representing a good candidate to explain the adverse reaction to ERT associated with the C1 protein inhibitor in patient II-5. We suggest measuring the C1 inhibitor level before ERT treatment in LOPD patients. 

Moreover, we demonstrated the important role of bone metabolism, in particular of vitamin D, as a key contributor in determining a worse phenotype and severe complications, including bone fractures. More studies are needed to confirm this association. However, we suggest that vitamin D serum levels and BMD should be monitored at regular intervals, and, if necessary, vitamin D should be supplemented. 

## 4. Materials and Methods

### 4.1. Patients

Patients were recruited at the Reference Centre for Neurological and Neuromuscular Rare Disease, University of Campania “Luigi Vanvitelli” (Figure 1A,B) [18]. 

For a better definition of the clinical heterogeneity of the LOPD patients (Table 1), we investigated 7 main functional areas divided into (1) motor function; (2) respiratory function; (3) body mass index; (4) bone metabolism; (5) immune response to ERT; (6) vascular anomalies; and (7) autophagy impairment.

All clinical data were collected at baseline, excepted for immune response to ERT, for which data were referred to a period after one year from the start of therapy. Motor function was assessed by MMT using the Medical Research Council (MRC) grading scale for different muscle groups, and the 6MWT was used to evaluate functional endurance during prolonged ambulation. The GSGC scale was performed as a measure of global motor disability. Respiratory assessment included spirometry for evaluation of FVC, considering as normal value FVC > 80% of predicted, and measurement of Δ-FVC, defined as drop of FVC from stand up to supine position (Δ-FVC > 20% is suggestive of restrictive pattern), according to the American Thoracic Society standards [20]. BMI was measured to provide an estimate of body fat in our patients. We defined a status of normal weight (19.5–24.9 kg/m^2^), overweight (25–29.9 kg/m^2^), obesity class I (30–35.9 kg/m^2^), obesity class II (36–40 kg/m^2^) and obesity class III (>40 kg/m^2^), according to WHO recommendation for Caucasian subjects [71]. Vitamin D serum levels (normal values: >30 ng/mL) were measured and the BMD of the lumbar-sacral tract, femoral neck and total-body (T-score normal value > −1) was evaluated by computerized bone mineralometry (MOC); both parameters were used for assessment of bone metabolism. History of bone fractures was considered. Basilar artery dolichoectasia was detected by brain MRI, and structural cardiac valvular defects were assessed by M-MODE/2D/Color-doppler cardiac ultrasound. Immunity state was determined using IgG and IgE antibodies against glucosidase alpha (anti-rhGAA); an adverse reaction to ERT was taken in account. After informed consent, muscle biopsy was obtained from all patients. Specimens from each biopsy were processed according to standard procedures for histology and biochemistry studies. Measurement of percentage of vacuolated fibres on muscle samplings, performed by optical microscopy on 3 fields at 20× on ATP pH 9.4 staining, and quantification of lymphocytes containing PAS-positive granules on blood smears [72] were carried out as indicators of impaired autophagy, together with assessment of GAA activity on DBS. All DBS were performed at the diagnostic service of Metabolic Laboratory, Centre of Diagnostics (Hamburg, Germany) by Dr. Zoltan Lukacs. The activity value of GAA was measured at pH 3.8, pH 7.0 and after specific inhibition. Normal GAA activity was considered between 1.86 and 21.9 mol/h/L. Beta-galactosidase activity was used as the internal control. Finally, venous blood samples were taken from patients for genomic DNA extraction and genetic analysis.

### 4.2. WES Analysis

WES was performed on 11 affected siblings (family 1: II-4, II-5, II-7, II-8, II-9, II-12, II-13; family 2: II-1, II-3, II-4, II-5). Exonic regions were enriched using the SureSelect All Exome kit V6 (Agilent Technologies, Santa Clara, CA, USA) based on DNA fragmentation and capture. Exomes were barcoded and sequenced at the Theragen Etex Bio Institute (Suwon, Korea), using the HiSeq2000 platform (Illumina, San Diego, CA, USA).

The alignment of the 100-bp paired-end reads to the human reference genome was performed by using the Burrows Wheeler Aligner (BWA) MEM, v0.7.542. After removal of duplicate reads through the Picard Mark Duplicates command (with standard options), we called the single nucleotide variants (SNVs) and insertions/deletions (indels) for all samples using HaplotypeCaller (BP_RESOULTION option) and GenotypeGVCFs in Genome Analysis Toolkit (GATK), v3.5-0-g36282e4, following the manufacturer’s best practice guidelines (available at https://software.broadinstitute.org/gatk/best-practices/ (accessed on 5 March 2021)) [73]. Variants with a Minor Allele Count (MAC) = 0, number of alternative alleles ≠2 and call rate <95% were also filtered out, as well as samples with identical-by-descent (IBD) sharing and sex mismatches, and samples with a call rate <90%. Variants passing quality control were annotated to genes (within 10 kb from transcription start/stop site) through ANNOVAR [74]. Variant annotation contained information concerning variant type, MAF in the general population and predictions of the variant’s effect on gene function. MAF was annotated in the National Heart, Lung, and Blood Institute (NHLBI) Grand Opportunity (GO) Exome Sequencing Project ESP6500si-v2 (European American and African American population) (http://evs.gs.washington.edu/EVS/ (accessed on 5 March 2021)), 1000 Genomes Project [AFR (African), AMR (Admixed American), EAS (East Asian), EUR (European), SAS (South Asian), http://www.1000genomes.org/ (accessed on 5 March 2021), Exome Aggregation Consortium (ExAC), EUR, non-Finish European population (NFE), AFR, SAS, EAS and AMR] (http://exac.broadinstitute.org/ (accessed on 5 March 2021)). SIFT (http://sift.bii.a-star.edu.sg/www/SIFT_seq_submit2.html/ (accessed on 5 March 2021)) PolyPhen2 (http://genetics.bwh.harvard.edu/pph2/ (accessed on 5 March 2021)) and CADD (cadd.gs.washington.edu/ (accessed on 5 March 2021)) were used to assess the deleterious effects of the identified variants.

Functional prioritization of the candidate genes was performed by STRING database analysis. The entire list of 589 candidate genes was analysed with the STRING database to search for protein–protein interaction (PPI) networks and for metabolic pathways enrichment. We found a significant PPI enrichment (*p* value = 1.7 × 10^−6^), which means that selected proteins have more interactions among themselves than what would be expected for a random set of proteins of similar size, drawn from the genome. In particular, we found a significant enrichment for genes related to skeletal muscle development and disease (*p* = 0.04) and in family 1 we identified a cluster of 18 gene products involved in calcium and bone metabolism (*p* = 0.03). Moreover, we analysed the metabolic pathways annotated by STRING to select the genes involved in the autophagy/lysosomal pathways as well as in immunity. Information were verified in OMIM and PubMed and selected variants were confirmed by direct sequencing, as already published [18].

### 4.3. Immunofluorescence Staining of Skeletal Muscle 

For immunofluorescence (IF) analysis, skeletal muscle cryosections (7 µm-thick) were fixed in pre-cooled acetone (−20 °C) for 5 min at room temperature (RT). Endogenous peroxidase activity was blocked by means of a 0.3% hydrogen peroxide and methanol solution for 5 min at RT. After washing, incubation with a blocking buffer (1 × PBS/5% horse serum) was performed for 1 h in a humidified chamber. Primary and then secondary antibody incubations were performed at 4 °C overnight and at RT for 2 h, respectively. Anti-LYAG (GTX109821, GeneTex, Irvine, CA, USA; 1:200), anti-LC3B (NB100-2220, Novus Biologicals, Littleton, CO, USA; 1:200), anti-p62/SQSTM1 (P0067, Sigma-Aldrich, St. Louis, MO, USA; 1:100), anti-LAMP2 (ab25621, Abcam, Cambridge, UK; 1:100), anti-FNIP2 (ab106611, Abcam, Cambridge, UK; 1:100), anti-RILP (ab140188, Abcam, Cambridge, UK; 1:50), anti-ADRP (PLIN2) (sc377429, Santa Cruz Biotechnology, Dallas, TX, USA; 1:100) primary antibodies and sheep anti-rabbit IgG-Cy3 (C2306, Sigma-Aldrich, St. Louis, MO, USA, 1:100), goat anti-rabbit FITC (F9887, Sigma-Aldrich, St. Louis, MI, USA, 1:100), sheep anti-mouse IgG-Cy3 (C2181, Sigma-Aldrich, St. Louis, MO, USA, 1:100) and sheep anti-mouse IgG-FITC (F2266, Sigma-Aldrich, St. Louis, MO, USA, 1:100) secondary antibodies were used. Slides were visualized with a Nikon Eclipse E800 photomicroscope. 

Muscle biopsies from a non-GAA vacuolar myopathy patient (CNT1) and from a non-myopathic orthopaedic patient (CNT2) were used as external controls, being available in the tissue bank of our department. 

### 4.4. Plasmids Preparation, Cell Cultures and Transfection

The wild-type (wt) expression vectors of *RILP* and *PLIN2* were purchased from the Addgene repository (EGFP-*RILP* catalogue: 110498; pEGFP-C1-*ADRP* (*PLIN2*) catalogue: 87161) and were used as the templates to introduce the *RILP* (c.G850A, p.G284S) and *PLIN2* (c.G616A, p.E206K and c.C925T, p.R309C) variants. Mutagenesis were carried out by the Quick Change II XL site-directed mutagenesis technique according to the manufacturer’s instructions (Agilent Technologies, Santa Clara, CA, USA). The whole coding sequences of wild-type and mutant plasmids were sequenced to confirm the mutagenesis and to exclude undesired mutations.

HeLa cells were cultured in Dulbecco’s modified Eagle’s medium (DMEM) supplemented with 10% FBS (Invitrogen, Thermo Fisher Scientific, Waltham, MA, USA) and 2% L-glutamine in a humidified atmosphere of 5% CO_2_ at 37 °C. 

In total, 500 nanograms of each vector were incubated with 1 microgram of Lipofectamine 2000 (Invitrogen, Thermo Fisher Scientific, Waltham, MA, USA) in Opti-MEM (Gibco, Thermo Fisher Scientific, Waltham, MA, USA) for 20 min; the mixture was added to HeLa cells (80,000 cells in 24 wells plate on coverslip) that were grown in complete medium for 20 h. Cells to be stained with anti-p62 and anti-LC3B antibodies were starved for 1 h in Hanks’ Balanced Salt solution (HBSS) before transfection. Twenty hours after transfection, cells were fixed with paraformaldehyde (PFA) 4% and stained with anti-LAMP2, anti-p62 and anti-LC3B antibodies. Briefly, incubation with blocking buffer (1×PBS/0.1% triton/5% serum) was performed for 15 min in a humidified chamber. Primary and then secondary antibody incubations (1 × PBS/0.1% triton/antibody) were performed at 4 °C overnight and at RT for 1 h, respectively. Anti-LAMP2 (ab25631, Abcam, Cambridge, UK, 1:100), anti-LC3B (NB100-2220, Novus Biologicals, Littleton, CO, USA, 1:200) and anti-p62/SQSTM1 (P0067, Sigma-Aldrich, St. Louis, MO, USA, 1:500) primary antibodies and anti-mouse IgG-Cy3 (C2181, Sigma-Aldrich, St. Louis, MO, USA, 1:200) and anti-rabbit IgG-Cy3 (C2306, Sigma-Aldrich, St. Louis, MO, USA, 1:200) secondary antibodies were used. Rhodamine-phalloidin (P1951, Sigma-Aldrich, St. Louis, Missouri, USA, 1:1000) and Hoestch H3570 (Thermo Fisher Scientific, Waltham, MA, USA; 1:1000) were used to visualize F-actin filaments and nuclei, respectively. Slides were visualized with a Nikon Confocal Microscope A1R and Nikon Eclipse Ni-E Microscope.

### 4.5. Expression Analysis of the Mutated FAM26Fc.525 + 2T > G Transcript

To assess the effect on splicing of the variant c.525 + 2T > G, located into the intron 2 of the FAM26F gene, we performed bioinformatics analysis by using the NetGene2 server (http://www.cbs.dtu.dk/services/NetGene2/ (accessed on 5 March 2021)), which produces neural network predictions of splice sites in human. We found that the c.525 + 2T > G variant completely disrupted (100% of probability) the canonical donor splicing site in intron 2.

To examine the expression pattern of the alternative transcript generated by the c.525 + 2T > G variant, an RT-PCR assay was performed using specific couples of oligonucleotides designed on exons 2 and 3 and on intron 2 of the gene. Blood RNA was isolated by using Tempus Spin RNA Isolation Kit (Thermo Fisher Scientific, Waltham, MA, USA). A total of 1 µg of total RNA, digested with DNasi RNasi free (Thermo Fisher Scientific, Waltham, MA, USA), was reverse transcribed with the Superscript III-First strand kit (Thermo Fisher Scientific, Waltham, MA, USA). Quantitative PCR (qPCR) reactions were performed in triplicate, using human *FAM26F* transcript-specific primers and ITaq Universal Sybr Green Supermix (Bio-Rad, Hercules, CA, USA) following the manufacturer’s directions. Results were normalized versus the expression of the beta-actin gene. The SD was calculated by using data of three different experiments. PCR products where directly sequenced on both strands using the Big Dye Terminator Ready Reaction Kit (Applied Biosystems, Foster City, CA, USA).

### 4.6. Statistical Analysis for Clinical Variables

Characteristics of the study population were presented using descriptive statistics. The mean and SD values were calculated for continuous variables, while frequencies were reported for categorical variables. Patients were classified as having a higher severity score (if moderate/severe or very severe score) or lower severity score (if mild or very mild score). Pearson correlation was used to explore the relationship between the severity score and clinical variables. A non-parametric test for independent samples (Mann–Whitney U) was used to compare the quantitative clinical variables (age, AAO, MMT-MRC, 6MWT, FVC, ΔFVC, BMI, VDD, BMD total body, BMD femoral neck, BMD lumbar-sacral and GSGC) in the two populations. Chi-squared tests were used to compare the qualitative variables (sex, BF, BAD, MVP, IgG-rhGAA and ERT-AE) between the two subgroups. A *p* value < 0.05 was considered statistically significant.

## 5. Conclusions

The findings of this study strongly support the hypothesis that disease severity and progression in LOPD patients is caused by the concomitant mutations of *GAA* and other autophagy genes. Of course, at this stage of advancement of our study, we cannot offer any improvement in practicing medicine; however, we believe it is important to report the two main results that emerge from this study. First is that the majority of the identified variants were located in autophagy-related genes, and second, some of these mutated genes were shared between the two LOPD families. Undoubtedly, the identification of potential modifier genes not shared by the majority of LOPD patients could cause great uncertainty on the interpretation of the clinical phenotype and of the ERT results collected in different series so far. However, at the molecular level, these findings offer a rational support to address additional genetic studies to identify in a larger cohort of LOPD patients the functional variants that could affect the autophagy pathway. A detailed molecular definition of Pompe disease would be essential to improve the treatment of the patients, in line with a personalized medicine approach.

## Figures and Tables

**Table 1:** Clinical data of the two LOPD Italian families.

ID	*GAA* Mut	Sex	Age	AAO	MMTMRC	6MWT	GSCG	FVC	Δ-FVC	BMI	VDD	BF	BMD T-Score TB/FN/LSV	BAD	MVP	IgG–rhGAA	DBS GAA	PPL	VMF
F1 II-4	p.R40X p.N882fs	F	67	50	79	297	12	79	35	22.20	6.64	YES	−2.9/−3/−3	YES	YES	1200	2.70	8	0.53
F1 II-5	p.R40X p.N882fs	F	65	53	82	225	12	62	46	28.00	11.6	NO	−1.3/−1.5/−2.6	YES	YES	6400	0.12	69	0.01
F1 II-7	p.R40X p.N882fs	M	61	42	76	324	7	98	17	28.10	28.4	NO	−1.1/−1.5/−0.5	YES	YES	1600	0.11	56	4.11
F1 II-8	p.R40X p.N882fs	F	59	47	86	325	9	90	18	33.10	18.5	YES	0.5/−0.7/−0.5	np	YES	1000	0.04	80	0.45
F1 II-9	p.R40X p.N882fs	M	58	38	74	254	9	83	20	21.40	12.7	NO	−0.8/−1.4/0.2	YES	YES	3200	0.09	48	11.18
F1 II-12	p.R40X p.N882fs	F	51	41	78	268	8	82	21	28.70	40.2	NO	1.1/2.2/0.6	YES	YES	1350	0.12	16	3.59
F1 II-13	p.R40X p.N882fs	M	50	36	75	254	6	105	18	20.10	41.3	NO	1.0/−0.8/−0.5	YES	NO	600	0.31	59	1.30
F2 II-1	c.-32-13T > G p.R375L	M	51	44	99	450	5	89	17	31.80	8.90	NO	−0.3/−0.5/0.9	NO	NO	1000	0.79	52	0.05
F2 II-3	c.-32-13T > G p.R375L	F	48	41	97	325	7	79	21	26.50	30.1	NO	0.4/0.1/1.3	NO	NO	1010	0.13	45	0.12
F2 II-4	c.-32-13T > G p.R375L	M	46	38	89	114	21	42	n.p.	39.18	8.4	NO	−0.8/−1.2/−0.5	NO	NO	850	0.78	58	5.28
F2 II-5	c.-32-13T > G p.R375L	F	41	29	83	10	26	36	n.p.	26.10	7.11	YES	−0.7/−2.1/1.9	NO	NO	900	0.98	63	15.31

F1: family 1; F2: family 2; F: female; M: male; Age: age at diagnosis; AAO: age at onset; MMT-MRC (Manual Muscle Testing—Medical Research Council) (%): normal value 100%; 6MWT (six minute walk test): normal value 480 ± 57 m (females) 580 ± 44 m (males); GSCG score (Gait, Stairs, Gower, Chair): normal score 4/27; FVC: forced vital capacity, normal value ≥80% of predicted; Δ-FVC (drop of FVC from sitting to supine); BMI (body mass index): normal range 18.5–24.9 kg/m^2^, overweight 25–29.9 kg/m^2^, obesity grade I 30–35.9 kg/m^2^, obesity grade II 36–40 kg/m^2^, obesity grade III > 40 kg/m^2^; VDD: vitamin D 25-OH dosage (normal range 30–100 ng/mL); BMD (bone mass density): total-body (TB), femoral neck (FN), lumbar-sacral vertebrae (LSV) T-Score, normal T-score value −1 g/cm^2^ and above, T-score values between −1 and 2.5 g/cm^2^ suggestive of osteopenia, T-score values −2.5 g/cm^2^ and below suggestive of osteoporosis; BF: bone fractures; BAD: basilar artery dolichoectasia and/or dilation of intracranial internal carotid; MVP: mild mitral valve prolapse; IgG-rhGAA (mg/dL): dosage of IgG anti rh-GAA after one year therapy. DBS GAA (mol/h/L): GAA enzyme activity on DBS, normal value 1.86–21.9 mol/h/L; PPL: percentage of PAS-positive lymphocytes (average of 30 fields at 40×); VMF: percentage of vacuole positive fibres (average of 3 fields at 20× on ATP pH 9.4); np: not performed.

**Table 2:** The severity index obtained adding the score of each clinical feature of a single LOPD patient.

	F1 II:4	F1 II:5	F1 II:7	F1 II:8	F1 II:9	F1 II:12	F1 II:13	F2 II:1	F2 II:3	F2 II:4	F2 II:5
AAO	1	1	2	2	3	2	3	2	2	3	3
MMT-MRC	3	2	3	2	3	3	3	1	1	2	2
6MWT	3	4	3	2	4	3	4	2	2	6	6
GSCG	2	2	1	2	2	1	1	1	1	3	4
FVC	1	2	0	0	0	0	0	0	1	4	4
Δ-FVC	4	4	2	2	2	3	2	2	3	4	4
BMI	0	1	1	2	1	1	0	2	1	3	1
VDD	2	2	1	2	2	0	0	2	0	2	2
BMD	2	1	1	0	1	0	0	0	0	1	1
BF	1	0	0	1	0	0	0	0	0	0	1
BAD	1	1	1	1	1	1	1	0	0	0	0
MVP	1	1	1	1	1	1	1	0	0	0	0
IgG-rhGAA	0	1	1	0	1	0	1	0	0	0	0
ERT-AE	0	1	0	0	0	0	0	0	0	0	0
Severity Index	21	23	16	17	21	15	16	11	11	28	28

Severity Index range: 1–47. AAO: age at onset; MMT-MRC (Manual Muscle Testing—Medical Research Council) (%): normal value 100%; 6MWT (six minute walk test): normal value 480 ± 57 m (females) 580 ± 44 m (males); GSCG score (Gait, Stairs, Gower, Chair): normal score 4/27; FVC: forced vital capacity, normal value ≥80% of predicted; Δ-FVC (drop of FVC from sitting to supine); BMI (body mass index): normal range 18.5–24.9 kg/m^2^, overweight 25–29.9 kg/m^2^, obesity grade I 30–35.9 kg/m^2^, obesity grade II 36-40 kg/m^2^, obesity grade III > 40 kg/m^2^; VDD: vitamin D 25-OH dosage (normal range 30–100 ng/mL); BMD (bone mass density): total-body (TB), femoral neck (FN), lumbar-sacral vertebrae (LSV) T-Score, normal T-score value −1 g/cm^2^ and above, T-score values between −1 and 2.5 g/cm^2^ suggestive of osteopenia, T-score values –2.5 g/cm^2^ and below suggestive of osteoporosis; BF: bone fractures; BAD: basilar artery dolichoectasia and/or dilation of intracranial internal carotid; MVP: mild mitral valve prolapse; IgG-rhGAA (mg/dL): dosage of IgG anti rh-GAA after one year of therapy; AE-ERT: adverse reaction to enzymatic replacement therapy.

**Table 3:** Clinical variables associated with a higher severity index score.

CLINICAL OUTCOMES	SEVERITY	N	Mean (SD)	*p*
6MWT (m)	VM/M	6	324.3 (69.5)	0.03
	M/S	5	180.0 (116.6)	
FVC (%)	VM/M	6	90.5 (9.73)	0.01
	M/S	5	60.4 (21.1)	
ΔFVC (%)	VM/M	6 3	18.6 (13.0)	0.02
	M/S		33.6 (18.8)	
VDD	VM/M	6 5	27.9 (11.4)	0.01
	M/S		9.29 (2.4)	
BMD femoral neck	VM/M	6 5	-0.20 (0.37)	0.03
	M/S		-1.80 (0.77)	
GSGC	VM/M	6 5	7.0 (1.41)	0.01
	M/S		16.0 (7.17)	

6MWT (m): six minute walk test (meters); FVC: forced vital capacity; Δ-FVC: drop of FVC from sitting to supine; VDD: vitamin D 25-OH dosage; BMD: bone mass density; GSCG score: Gait, Stairs, Gower, Chair; VM/M: very mild or mild phenotype; M/S: moderate/severe or very severe.

**Table 4:** Genes selected by functional prioritization in families 1 and 2.

Type of Analysis	Patient ID Family 1	Gene	Omim/RF	Inheritance	SKM Related Disease	A	I	M	CVM	O/BF	VD	Ob
Severity score > 20 F1 and F2	F1 and F2	*RILP*	nr	nr	nr	yes	yes	nr	nr	nr	nr	nr
	F1 and F2	*FNIP2*	nr	nr	nr	yes	yes	nr	nr	nr	nr	nr
	F2	*TRAPPC11*	LGMDR18	AR	yes	yes	yes	yes	nr	nr	nr	nr
BMI	F1 and F2	*PLIN2*	Obesity	nr	nr	yes	nr	yes	nr	nr	nr	yes
BMD/BF/VDD F1	II-4, II-5	*TRPV6*	HRPTTN	AR	nr	nr	nr	nr	nr	yes	yes	nr
	II-4, II-5, II-7, II-9, II-12, II-13 (II-8 only IRS1)	*IRS1*	NIDDM	RF	nr	yes	yes	yes	nr	yes	yes	yes
		*KL*	HFTC3	nr	nr	yes	yes	nr	nr	yes	yes	nr
		*TRPV5*	nr	nr	nr	nr	nr	nr	nr	yes	yes	nr
BMD/BF/VDD F2	II-5	*LRP4*	CMS17	AR	yes	yes	yes	yes	nr	yes	nr	yes
		*RUNX1*	FPDMM	AD	nr	yes	yes	yes	nr	yes	yes	nr
MVP F1	II-4, II-5, II-7, II-8, II-9, II-12	DCHS1*	MVP2	AD	yes	nr	nr	yes	nr	nr	nr	nr
AR-ERT F1	II-5	*FAM26F*	nr	nr	nr	nr	yes	nr	nr	nr	nr	nr

BMI: body mass index; BMD: bone mass density; BF: bone fractures; VDD: vitamin D deficiency; MVP: mild mitral valve prolapse; AR-ERT: adverse response to enzyme replacement therapy; SKM-related disease: skeletal muscle-related disease; A: autophagy; I: immunity; M: muscle related; CVM: cerebrovascular malformations; O: osteoporosis; BF: bone fractures; VD: vitamin D related; Ob: obesity related; nr: not related; HRPTTN: Hyperparathyroidism, Transient Neonatal; NIDDM: Diabetes Mellitus, Noninsulin-Dependent; HFTC3: Tumoral Calcinosis, Hyperphosphatemic, Familial, 3; CMS17: Myasthenic Syndrome, Congenital, 17; FPDMM: Platelet Disorder, Familial, with associated myeloid malignancy; MVP2: Mitral Valve Prolapse 2; CMD1BB: Cardiomyopathy, Dilated, 1BB; ARVD10: Arrhythmogenic Right Ventricular Dysplasia, Familial, 10; CMH26: cardiomyopathy, familial hypertrophic, 26; MPD4: Myopathy, Distal, 4; MFM5: Myopathy, Myofibrillar, 5; BTHLM1: Bethlem Myopathy 1; LGMDR18: Muscular Dystrophy, Limb-Girdle, Autosomal Recessive 18; RF: risk factor; AR: autosomal recessive; AD: autosomal dominant. DCHS1*: II-5, II-7 and II-12 were compound heterozygous for DCHS1 variants. FLNC*: patients II-4, II-5 and II-13 were compound heterozygous for the two FLNC variants.

**Table 5:** List of the rare and deleterious variants identified in the two families.

Type of Analysis	Family	Gene	avsnp147	Accession Number	Exonic Function	Nucleotide Change	AA Change	MAF Max	CADD Phred	SIFT	PolyPhen2
Severity score > 20	F1 and F2	*RILP*	rs61735419	NM_031430	NSV	c.G850A	p.G284S	0.04	27.4	T	D
	F1	*FNIP2*	rs148251675	NM_020840	NSV	c.T901C	p.S301P	0.02	23	T	T
	F2	*FNIP2*	rs62001914	NM_020840	NSV	c.C1653A	p.S551R	0.03	25	D	T
	F2	*TRAPPC11*	rs62617790	NM_021942	NSV	c.G2799C	p.Q933H	0.13	23	D	D
BMI	F1	*PLIN2*	rs759915698	NM_001122	NSV	c.C925T	p.R309C	0.00003	24	D	D
	F2	*PLIN2*	nr	NM_001122	NSV	c.G616A	p.E206K	na	27	D	D
BMD/BF/VDD	F1	*TRPV6*	rs778016744	NM_018646	NSV	c.G1886A	p.R629Q	0.000008	24	T	P
	F1	*TRPV5*	nr	NM_019841	NSV	c.C1894T	p.R632W	na	33	D	D
	F1	*KL*	rs9536314	NM_004795	NSV	c.T1054G	p.F352V	0.19	27	D	D
	F1 and F2	*IRS1*	rs1801278	NM_005544	NSV	c.G2911A	p.G971R	0.08	24	T	P
	F2	*LRP4*	rs72897663	NM_002334	NSV	c.A1501C	p.N501H	0.07	21	T	T
	F2	*LRP4*	rs117936904	NM_002334	NSV	c.T5165A	p.L1722H	0.02	29	D	D
	F2	*RUNX1*	nr	NM_001754	NSV	c.G190A	p.G64S	na	23	T	P
MVP	F1	DCHS1*	rs35599968	NM_003737	NSV	c.G8480C	p.R2827P	0.10	20	T	T
	F1	DCHS1*	rs376287018	NM_003737	NFins	c.99_100insCTG	p.G34insLG	0.10	nr	nr	nr
AR-ERT F1	F1	*FAM26F*	rs117361304	NM_001010919	splicing	c.525 + 2T > G	nr	0.09	25.4	nr	nr

BMI: body mass index; BMD: bone mass density; BF: bone fractures; VDD: vitamin D deficiency; MVP: mild mitral valve prolapse; AR-ERT: adverse response to enzyme replacement therapy; AA change: amino acid change; MAF (Minor Allele Frequency) max: the highest frequency value reported in public databases is reported (aESP6500si-v2 (European American and African American population), 1000 Genomes project (AFR, AMR, EAS, EUR, SAS), ExAC browser (NFE, AFR, SAS, EAS and AMR)); CADD phred: Combined Annotation Dependent Depletion, a value ≥20 corresponds to those variants which are predicted to be amongst the 1% most deleterious variants in the genome; nr: not reported; D: deleterious; T: tolerated; P: probably damaging; DCHS1*: II-5, II-7 and II-12 were compound heterozygous for DCHS1 variants.

**Table 6:** Genotype distribution of the *VDR* and *GC* polymorphisms associated to vitamin D levels.

					*VDR*			*GC/DBP*
ID	Sex	Age	AAO	VDD (ng/mL)	rs2228570 c.T2C p.M1T FokI Variant	rs7975232 G/T ApaI	rs731236 c.T1056C p.I352I TaqI Variant	rs4588 c.C1307A p.T436K
F1 II-4	F	67	50	6.64	CC	GT	TC	CC
F1 II-5	F	65	53	11.6	CC	GG	TT	CC
F1 II-7	M	61	42	28.4	CC	GG	TT	CC
F1 II-8	F	59	47	18.5	CC	GT	TC	CC
F1 II-9	M	58	38	12.7	TC	GT	TC	CC
F1 II-12	F	51	41	40.2	TC	TT	CC	CC
F1 II-13	M	50	36	41.3	CC	GT	TC	CC
F2 II-1	M	51	44	8.90	CC	TT	TC	CC
F2 II-3	F	48	41	30.1	CC	TT	TT	CA
F2 II-4	M	46	38	8.4	CC	TT	CC	CC
F2 II-5	F	41	29	7.11	CC	TT	TC	CC

AAO: age at onset; VDD: vitamin D dosage; VDR: vitamin D receptor; GC/DBP: vitamin D-binding protein.

## Data Availability

The datasets analyzed during the current study are available from the corresponding author on reasonable request.
